# Seeing Suppresses Haptic Pleasure While Perceiving Contemporary Art

**DOI:** 10.1177/2041669520932948

**Published:** 2020-06-30

**Authors:** Magdalena Szubielska, Ewa Niestorowicz

**Affiliations:** The John Paul II Catholic University of Lublin, Institute of Psychology, Lublin, Poland; Maria Curie-Skłodowska University, Institute of Pedagogy, Lublin, Poland

**Keywords:** contemporary art, haptics, empirical aesthetics, visuo-haptic interactions

## Abstract

To date, haptic aesthetic processing has been tested outside the field of real works of art. By providing the context of a contemporary art exhibition designed to be touched, we studied haptic pleasure towards artworks. In line with our hypothesis, seeing affected the evaluation of haptic pleasure which was higher in the blindfolded-tactile than visuo-tactile condition. Thus, seeing seems to impede the tactile processing of artworks.

Touching artworks can be considered as a kind of guilty pleasure. In most museums and art galleries, touching the exhibits is strictly forbidden, although many visitors have a desire for haptic contact with art (Chatterjee, 2008). The viewers only occasionally have the opportunity to explore original works of art by hand while contemplating sculptures available in public space (Muth et al., 2019), interactive installations (Szubielska et al., 2019), or artworks designed with the thought of an audience with visual impairment (Szubielska, 2018). Perceiving visual arts mainly through sight to some extent explains why studies in the field of the psychology of art perception are predominantly vision-centred (Augustin & Wagemans, 2012). Moreover, an interiorised rule towards banning the touching of artworks may explain the decrease in aesthetic appreciation in adult viewers who were allowed to perceive art both visually and haptically in comparison with viewers who have only seen works of art (Sánchez Clemente, 2017). The solution to the cognitive dissonance likely to be created by touching real works of art could be to consider them less valuable (because precious works of art cannot be touched). Thus, without giving explanations as to why, exceptionally, artworks may be touched, the aesthetic evaluation may be biased by a belief related to the reduced value of a work of art that is available by touch.

To date, only a few studies have focused on haptic aesthetic processing. This research concerned the reception of a convex collage (Muth et al., 2019), evaluation of the surface material of products (Jakesch et al., 2011), or stimuli specially created for the study (Soranzo et al., 2018). Although Carbon and Jakesch (2013) developed an elegant model of haptic aesthetic processing, to our knowledge, no research on the reception of works of art has so far been conducted within its theoretical framework. In the current study, we aimed to fill this gap and further extend a non-vision-centred view on empirical aesthetics to the area of artworks. Because tactile stimuli are processed more efficiently in only-tactile than visuo-tactile conditions (Klein, 1977; Posner et al., 1976; see Spence, 2007 for review on how vision modulates haptic perception) and the evaluation is the last, most demanding stage of haptic aesthetic processing (Carbon & Jakesch, 2013), we predicted that self-rated haptic pleasure is higher when visitors perceive artworks haptically, compared with the multimodal (visual and tactile) cognition condition.

To test our hypothesis, we asked artistically untrained participants (*N* = 91, 68 female, aged: *M* = 21.96, *SD* = 1.80) to acquaint themselves with the “Touch of art” exhibition. The exhibition, located in the gallery area within the Faculty of Fine Arts building on the Maria Curie-Skłodowska University campus, was originally designed for viewers with visual impairments (the participants were aware of this). All exhibited works were allowed to be freely touched, and their labels were covered for the duration of the study. The audience perceived the works of art in two different ways: either in the haptic domain (being blindfolded and having a sighted peer guide) or by seeing and touching them. After reception of each of the 16 works of art (see Figure 1) in unlimited time, the participants assessed (on 8-degree scales) to what extent the works were pleasant to the touch (haptic pleasure was the dependent variable in this study), subjectively understandable, and liked (controlled variables).

**Figure 1.**
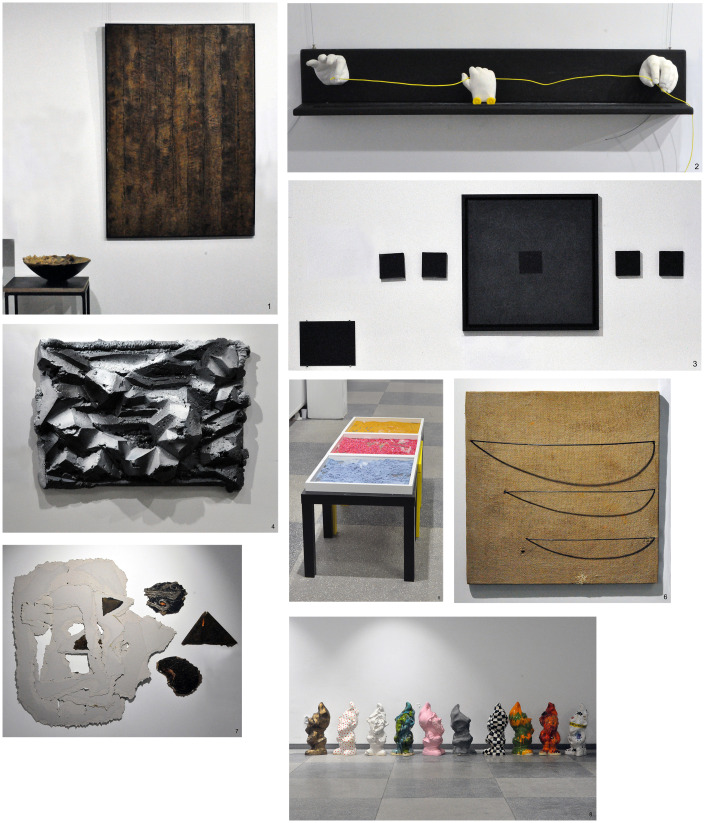

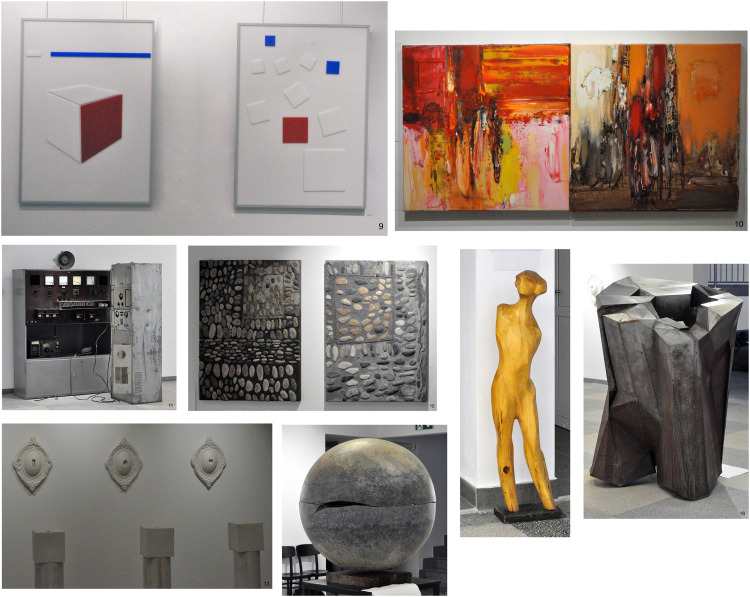
Order of Artworks’ Presentation (continued overleaf).

The preliminary analysis showed that the way the exhibition was perceived did not affect either understanding, *t*(89) = 1.17, *p* = .245, nor liking, *t*(89) = .88, *p* = .380. Haptic pleasure, subjective understanding, and liking were strongly correlated with each other (*r* values ranged between .70 and .83, all *p*s < .001). Stepwise regression analysis showed that the haptic pleasure was best predicted by liking (included in the first model, *R*^2^ = .69; the second model additionally included the variable of understanding, but the change of *R*^2^ was only .02). An analysis of covariance with the perceptual condition as the between-participant variable and liking of the artworks as a covariate yielded that additional visual perception significantly reduced haptic pleasure, *F*(1, 88) = 5.69, *p* = .019, η_p_^2^ = .06 (the tactile condition: *M* = 4.77, *SD* = 0.84; the visual and tactile condition: *M* = 4.67, *SD* = 0.75).

Although the found effect was very small and subsequent research with a beforehand power analysis provided is needed to validate the findings, it seems that sight may suppress the haptic pleasure coming from touching art. The contextual information may shape visitors’ certain haptic expectations (Carbon & Jakesch, 2013), and seeing seems to be a major source of context in multisensory perception. Therefore, probably in the visuo-haptic condition, participants rated haptic pleasure in relation to sight-based expectations, and the artworks did not feel as expected. It is also possible that visual aspects of the artworks drew the viewer’s attention and at the same time hindered haptic information processing (Klein, 1977; Posner et al., 1976; Spence, 2007).

Hence, exposing artworks illuminated with muffled light or unlighted may increase the likelihood of experiencing haptic aesthetic pleasure when touching art. Hopefully, some innovative curator will soon try out this idea.

## Supplementary Material

Supplementary material
